# Improvements for Therapeutic Intervention from the Use of Web Applications and Machine Learning Techniques in Different Affectations in Children Aged 0–6 Years

**DOI:** 10.3390/ijerph19116558

**Published:** 2022-05-27

**Authors:** María Consuelo Sáiz-Manzanares, Raúl Marticorena-Sánchez, Álvar Arnaiz-González

**Affiliations:** 1 Research Group DATAHES, Departamento de Ciencias de la Salud, Facultad de Ciencias de la Salud, Universidad de Burgos, Pº Comendadores s/n, 09001 Burgos, Spain; 2 Research Group ADMIRABLE, Departamento de Ingeniería Informática, Escuela Politécnica Superior, Universidad de Burgos, Avd. de Cantabria s/n, 09006 Burgos, Spain; rmartico@ubu.es (R.M.-S.); alvarag@ubu.es (Á.A.-G.)

**Keywords:** early care, web application, machine learning techniques, precision therapeutic program, personalized intervention, disabilities

## Abstract

Technological advances together with machine learning techniques give health science disciplines tools that can improve the accuracy of evaluation and diagnosis. The objectives of this study were: (1) to design a web application based on cloud technology (eEarlyCare-T) for creating personalized therapeutic intervention programs for children aged 0–6 years old; (2) to carry out a pilot study to test the usability of the eEarlyCare-T application in therapeutic intervention programs. We performed a pilot study with 23 children aged between 3 and 6 years old who presented a variety of developmental problems. In the data analysis, we used machine learning techniques of supervised learning (prediction) and unsupervised learning (clustering). Three clusters were found in terms of functional development in the 11 areas of development. Based on these groupings, various personalized therapeutic intervention plans were designed. The variable with most predictive value for functional development was the users’ developmental age (predicted 75% of the development in the various areas). The use of web applications together with machine learning techniques facilitates the analysis of functional development in young children and the proposal of personalized intervention programs.

## 1. Introduction

Nowadays, technology has provided the health sciences with tools to help diagnose and intervene in different afflictions. These tools are related to technological advances and the use of artificial intelligence (it can be defined as the use of machines that simulate the human way of processing information) and machine learning techniques (they are either prediction or classification algorithms). These will be extremely useful for the production of personalized treatment plans. Once integrated in software applications that make these technologies easy to use, the combination will be invaluable help for healthcare professionals. In addition, it will ensure both primary and secondary prevention for various pathologies. This method of working is called precision medicine or precision psychology. The former is principally related to an approach for the prevention or treatment of illnesses in which one must consider the individual variability of a person’s genes, environment, and lifestyle [1]. Precision medicine basically finds accurate predictors of results, driving personalized clinical treatment. Research into it began around 20 years ago [2]. Nowadays, these techniques are used very successfully for diagnosing and treating cancer [3] and in immune therapies [4,5]. To do that, it is necessary to determine prognostic biomarkers, and based on them, apply the therapy that is most likely to be more effective [6]. The machine learning techniques that are used are supervised learning (prediction) and unsupervised learning (clustering) [7].

**Figure 1:**
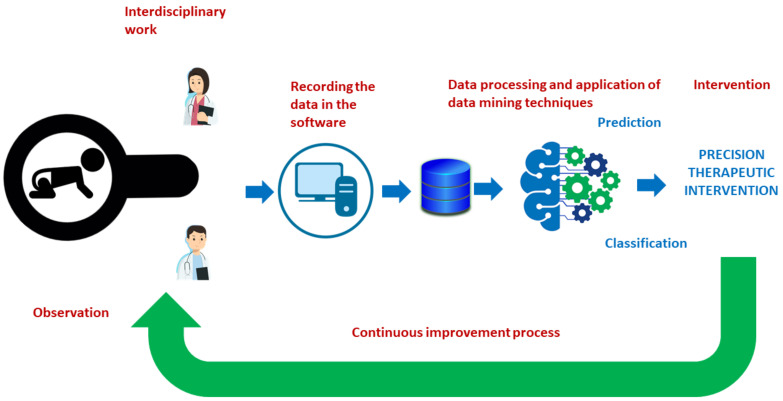
The diagnostic and intervention process using technological resources and machine learning techniques in early care.

This area of activity can be transferred to the context of precision psychology [8], which seeks patients’ characteristics that facilitate diagnosis via prediction [9] as well as the most effective therapeutic approach depending on the profiles found. Machine learning techniques are being used in interventions for phobias [10] and in depressive disorders [11], applying self-instructional therapies and personalized cognitive therapies [11,12]. In addition, these tools are beginning to be implemented in the field of early care for children with a variety of conditions [13]. This is particularly important for diagnosis and intervention for different developmental pathologies in infancy and childhood, and also leads to more precise interventions [14]. Human developmental progress is a dynamic process and understanding it must be based on systematic observation [14]. The first few years of life (0–6 years old) see the development of perception and motor skills, cognitive, communication and language skills, and socio-emotional skills. A range of healthcare professionals work in this field (neonatologists, neurologists, pediatricians, physical therapists, psychiatrists, physiologists, physiotherapists, occupational therapists, speech therapists, etc.). They need computer-based tools that allow them to make effective differential diagnoses, as a good diagnosis is the beginning of a good intervention [9,15,16]. Computer-based tools, together with artificial intelligence and machine learning techniques integrated in web applications, will assist this process of personalizing interventions and thus enhance therapeutic success [9]. In addition, using these techniques will improve the cost-effectiveness of both personal and material resources, and will improve the prognosis in various pathologies. In recent years, these technologies have been used via cloud-based applications which make them much easier for early care professionals to use [17,18]. An example of this procedure of working is given in Figure 1.

### Creation of Prototypes for Evaluation and Therapeutic Intervention in Different Developmental Issues

The creation of prototypes for recording and analyzing developmental progress is extremely important for detecting problems. However, it is a laborious process requiring the clear definition of the developmental areas to be explored and the items defining observable behaviors. These must be based on validated protocols related to development in the various areas. In addition, the type of measuring scale to be applied for the evaluation of each behavior must be defined. Likert-type scales are advisable for this because they allow a continuous range in the evaluation beyond the all-or-nothing of dichotomous scales. This is important in the assessment of development as using Likert-type scales allows the patient’s progress to be tracked longitudinally. One solution is the use of computer applications that include algorithms that allow the assessment and interpretation process to be automated, as well as the results to be visualized. The use of these tools will be fundamental for the healthcare professionals responsible for diagnosis (neurologist, neuropediatrician, pediatrician, psychologist, etc.) and those responsible for intervention (occupational therapist, speech therapist, physiotherapist, etc.) and follow-up of patients with early developmental issues. Finally, it is important to remember that prevention is one of the objectives recognized by UNICEF in the rights of the child [19].

The use of machine learning and artificial intelligence techniques is being pioneered in disciplines such as psychology and neuroscience. Moreover, their use is considered to have a promising future for the advancement of diagnosis and therapeutic intervention [20,21]. However, a limited scope and the need to increase studies in this field have been detected [21].

Considering the previous references, in this study, we are going to analyse how an assessment and therapeutic intervention process could be carried out to be applied to users with different developmental impairments in the 0–6 age group. The collection of information will be carried out through a web application and machine learning techniques will be used to analyse the results of the observation.

Based on the above, the objectives of this study were:

(1) To develop a web application based on cloud technology to obtain personalized therapeutic intervention programmes for children from 0 to 6 years of age with different developmental disorders (eEarlyCare-T).
(2) Conduct a pilot study to test the usability of the web application (eEarlyCare-T).
(3) Apply Machine Learning techniques to analyze the results.

## 2. Materials and Methods

### 2.1. Participants

We carried out a pilot study to evaluate the effectiveness of the eEarlyCare training web application (eEarlyCare-T) using a sample of 23 children (5 girls and 18 boys), diagnosed with various developmental problems according to the Diagnostic and Statistical Manual of Mental Disorders, Fifth Edition (DSM-5) [22]. The diagnoses were performed by professionals working in neurology, pediatrics, and psychology in early care services as part of early detection programs for children aged 0–6 years old. The children attended special schools where they received general stimulation, speech therapy, physiotherapy, and occupational therapy. Table 1 gives a description of the sample. Participation in the pilot study was voluntary within a specific center for children with different disabilities. Prior to the study, we obtained authorization from the Bioethics Committee of the University of Burgos, followed by authorization from those responsible for the institution, after which the children’s families were informed and written informed consent was obtained. The therapists who attended the children also signed a consent form and used the eEarlyCare web application.

### 2.2. Procedure

(a) The eEarlyCare project: Data recording and treatment module.

Since 2016, the University of Burgos, together with the government of Castilla y León and European funds (FEDER), have encouraged the appraisal and commercialization of research results through the selection of projects which provide value through prototypes applied to practical intervention. The eEarlyCare project was first selected in 2018. In this phase, a computer application was developed for recording and automating the grading of a measurement scale of functional abilities for 0–6 years-old [17]. This project presented a proposed prototype for recording and automatically interpreting the evaluation of abilities in 11 functional areas (Food Autonomy, Personal Care and Hygiene, independently dresses and undresses, Sphincter control, Functional mobility, Communication and Language, Resolution of tasks in social contexts, Interactive and symbolic play, Daily routines, Adaptive behavior, and Attention) in development ages 0–6 years [23]. The application was based on the Scale for the Measurement of Functional Abilities in 0–6 years old (SFA) [24].

(b) The prototype was created via the following steps:

Step 1. An observation protocol was added to a web application that included a marking or grading system. The detected deviations from the expected development age are based on the subject’s chronological age. It was developed as follows:

Step 1.1. Studying the scales and protocols measuring development of functional abilities in ages 0–6 years old (“Scale of psychomotor development of early childhood Brunet-Lézine-Revised” [25], “Battelle Developmental Inventory” [26], and “The Pediatric Evaluation of Disability Inventory” [27]). The protocol was validated in two pilot studies [13,17].

Step 1.2. Establishing a marking or grading system. Each behavioral analysis item was associated with a developmental age based on the reference of progressive acquisition from the previously validated scales. The subject’s developmental achievement was measured on a Likert-type scale from 1 to 5 (non-acquisition to complete acquisition of the functional ability) [17].

**Figure 2:**
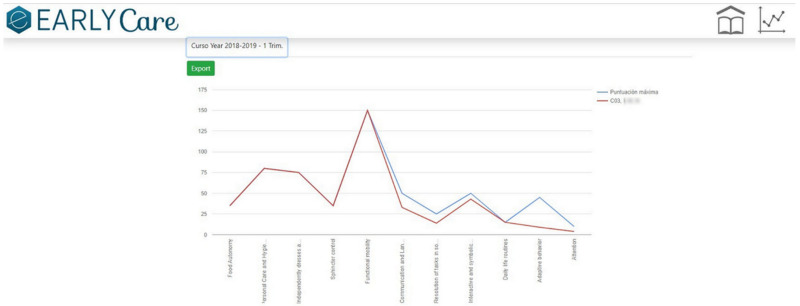
A patient’s functional ability development profile.

**Figure 3:**
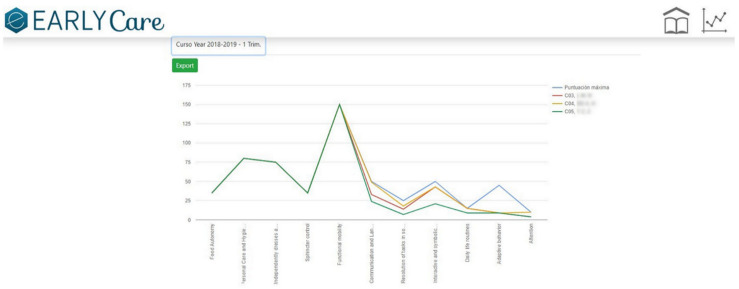
Functional ability development profile for a group of patients.

**Figure 4:**
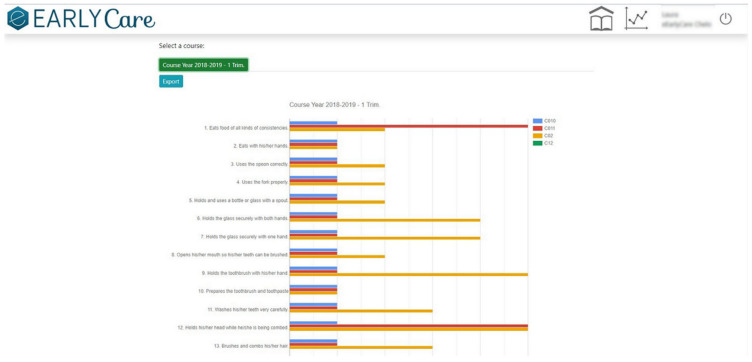
Levels of a patient’s development in each of the abilities that measure each of the behaviors include into each functional area.

(c) Application of data visualization techniques. A module was created within the eEarlyCare web application [23] allowing comparison between expected developmental progress and observed development in the various areas. This module makes it easy to detect the most problematic areas for a specific child or group of children, examples of which are given in Figure 2 and Figure 3. The blue line indicates normal development for the chronological age, the red line indicates the observed developmental progress.
(d) The data were analyzed using machine learning techniques, both supervised learning (prediction) and unsupervised learning (clustering). In order to do that, the eEarlyCare web application allows data to be exported in .xls format. This format can be read by statistical data analysis software such as SPSS [28] and visualization programs such as Orange [29].
(e) The results provide a development profile (it refers to development in different developmental areas) and, based on that, a personalized therapeutic intervention plan (refers to the elaboration of therapeutic personalized intervention programs to the developmental profile found in each user) for each patient is designed. The intervention plan prioritizes the most affected functional areas, and within those, the items referring to behavior (see Figure 2, Figure 3 and Figure 4).
(f) eEarlyCare-T web application includes the Therapeutic program: depending on the profile found, guidelines are given for the development of a specific therapeutic intervention program.

**Figure 5:**
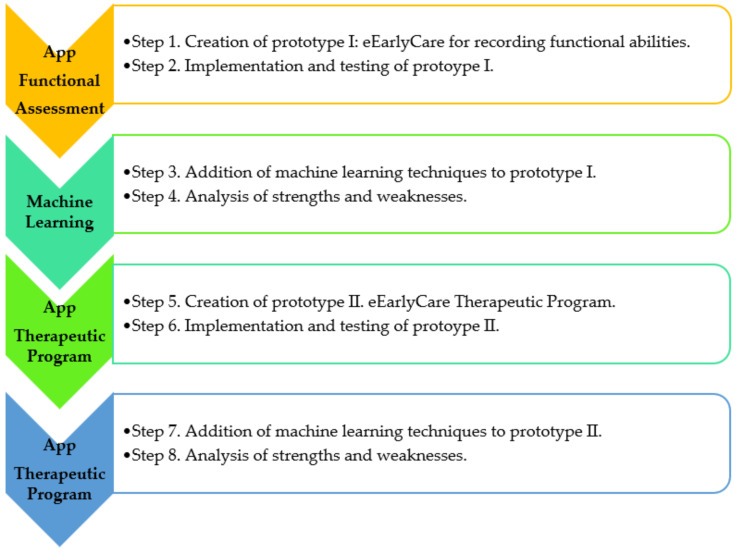
Functional and data requirements guiding the development of the eEarlyCare-T web application. Note. The results of the step 1 to 4 can be found in the publications [13,15,17].

First of all, a therapeutic intervention program that enables the acquisition of functional abilities (in the 11 development areas [30,31,32,33,34]) was created. Secondly, a web application that makes it easier for staff creating personalized programs (based on a children’s functional development profile) was developed. Figure 5 summarizes the most relevant steps of the eEarlyCare-T web application design.

### 2.3. Design

A pre-experimental design was used [35].

### 2.4. Statistical Analysis

We used descriptive statistics along with unsupervised machine learning techniques clustering k-means and hierarchical as well as a fixed-effects ANOVA (cluster type) and supervised machine learning techniques (Multiple Linear Regression). We used the SPSS v.25 [28] statistical package and Orange v.3.30 [29] data visualization software.

## 3. Results

**Figure 6:**
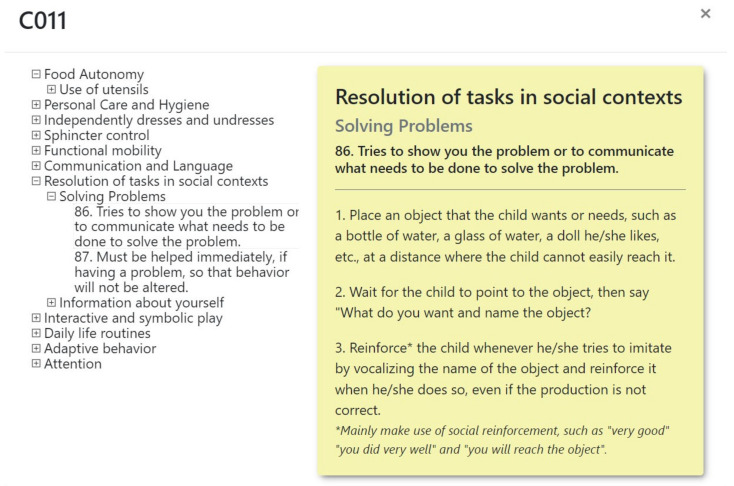
Example of guidance cards for implementation of a functional ability.

In relation to the first objective, we produced the data recording and treatment module of the eEarlyCare-T web application. This module included the recording, treatment, and interpretation of the patients’ assessment results in the 11 functional areas. The results can be found in different studies [15,17]. The eEarlyCare-T: Recording and treatment module facilitates diagnosis by professionals at early ages (0–6 years) through the visual interpretation of the results (see Figure 2, Figure 3 and Figure 4). Then, we elaborated the eEarlyCare Therapeutic Program module. This module guides the creation of personalized therapeutic intervention programs. We established the following milestones:

Milestone 1. Creation of therapeutic guidance for intervention in each of the functional abilities indicated in the eEarlyCare-T web application. To create these guides, we used materials including the Portage Guide [36], the Mentalist Stimulation Program in Early Infancy [37] and the Ability Development Program in small children [38]. An example is given in Figure 6.

**Figure 7:**
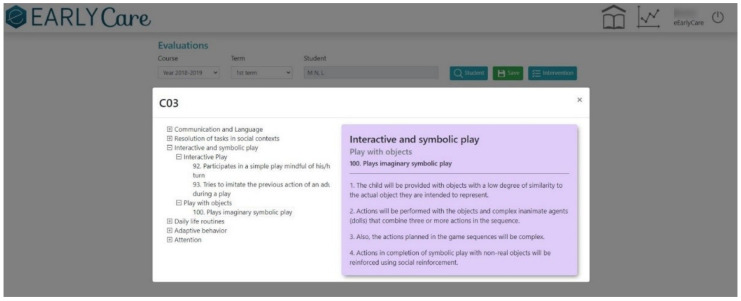
Example of a proposal for a personalized therapeutic intervention in the area interactive and symbolic play with eEarlyCare-T.

Milestone 2. Creation of the Therapeutic Intervention Program in the eEarlyCare-T web application. The program is presented in English and Spanish and includes personalized guidance for therapeutic intervention based on each child’s profile. As an example, Figure 7 shows the therapeutic guidance for intervention in the functional area of Interactive and Symbolic Play according to the developmental profile found. In addition, multimedia file 2 shows the “Therapeutic Intervention Program” in the eEarlyCare-T web application (see Supplementary Materials Video S1 to see how the web application functions).

Then, to address the second objective, we use an ad hoc questionnaire based on the UEQ-S short version [39] completed by therapists. The usability scale consisted of 19 questions: The first 5 related to informed consent and data about age, educational qualifications, working situation, and gender. Items 6–15 were closed questions asking about the usability of the application via a Likert-type scale from 1–5, and items 16–19 were open questions assessing the functionality of the application. However, the results should be taken with caution as only 4 therapists participated in this pilot study. The usability questionnaire used can be seen in Supplementary Materials Table S1.

We analyzed the usability of the web application eEarlyCare-T through the responses of the therapists involved in the pilot study. The results of the closed response items are given in Table 2, and Table 3 shows the results to the open response items, both from the Short Version of UEQ-S [39].

Specifically, Table 3 shows the results of the answers at the open questions of the usability questionnaire (items 16 to 19).

Then, in order to test objective 3, machine learning techniques were applied. Specifically, to find groups in the data, we used *k*-means, a clustering technique (unsupervised learning). We found three clusters in relation to development in the 11 functional areas, and we applied *k* = 3 (see Table 4).

We found significant differences between the clusters in the functional areas of Food autonomy [*F*(2,20) = 12.34, *p* = 0.00], Personal care and hygiene [*F*(2,20) = 30.98, *p* = 0.001], Independently dresses and undresses [*F*(2,20) = 80.64, *p* = 0.00], Sphincter control [*F*(2,20) = 37.76, *p* = 0.001], Functional mobility [*F*(2,20) = 65.18, *p* = 0.001], Interactive and symbolic games [*F*(2,20) = 8.72, *p* = 0.002], and Daily life routines [*F*(2,20) = 14.28, *p* = 0.001], but not in Communication and Language [*F*(2,20) = 3.10, *p* = 0.07], Resolution of tasks in social contexts [*F*(2,20) = 2.80, *p* = 0.09], Adaptive behavior [*F*(2,20) = 0, *p* = 0.99] or Attention [*F*(2,20) = 2.36, *p* = 0.12] at a 95% confidence level.

Following the reviewer advice, the elbow method has been used for finding the best “k” value for k-means clustering. In the following picture is shown the sum of squared distances of samples to their closest cluster center (in y axis) for each k value from 1 to 10 (x axis). Subsequently, we produced a cross table between the value of the assigned cluster for each participant and the variable primary diagnosis (see Table 5).

**Figure 8:**
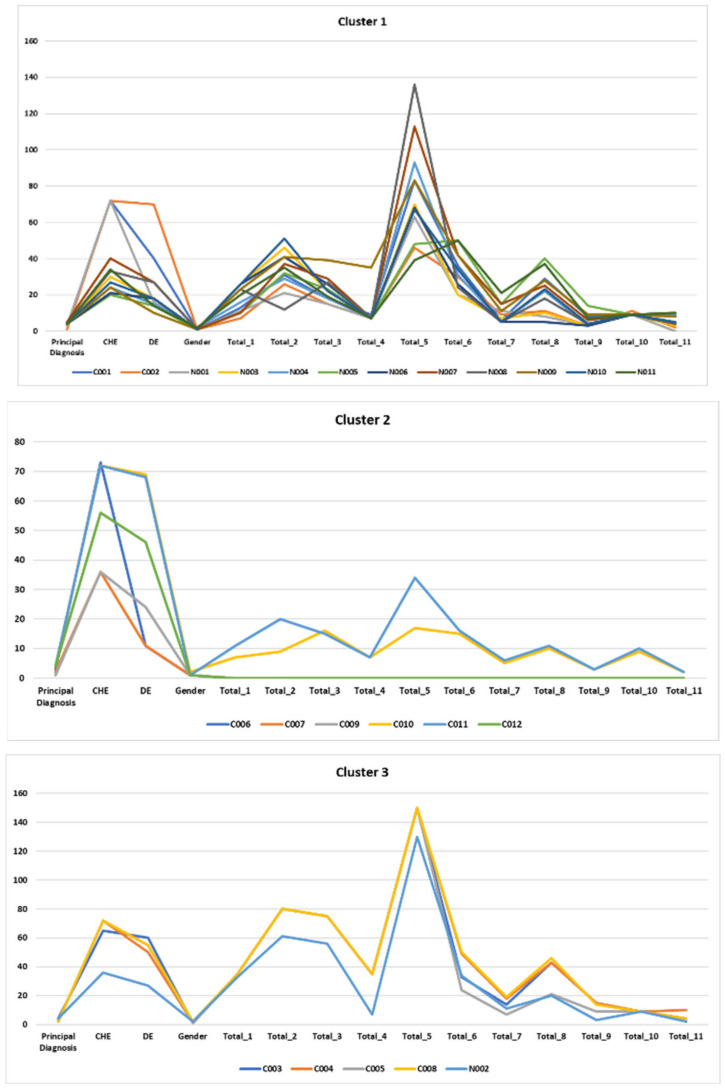
Relationship of the variables studied with respect to the grouping of the participants in the three clusters. Note. CHE = Chronological age; DE = Developmental age; Total_1 = Food autonomy; Total_2 = Personal care and hygiene; Total_3 = Independently dresses and undresses; Total_4 = Sphincter control; Total_5 = Functional mobility; Total_6 = Communication and language; Total_7 = Resolution of tasks in social contexts; Total_8 = Interactive and symbolic games; Total_9 = Daily life routines; Total_10 = Adaptive behavior; Total_11 = Attention.

Table 5 shows that the assigned diagnostic category does not correspond to the cluster the patient belongs to except for diagnosis 5; in addition, the contingency coefficient was 0.63. This is an important aspect, as the diagnosis is often based on secondary developmental problems such as motor problems, and at young ages this type of development is directly related to the evaluation of functional development. Figure 8 gives a visualization of the behavior of subjects classified in the clusters in the various areas of functional development, in addition to the variables chronological age, developmental age, gender, primary diagnosis and secondary diagnosis regarding the distribution of the three clusters.

**Figure 9:**
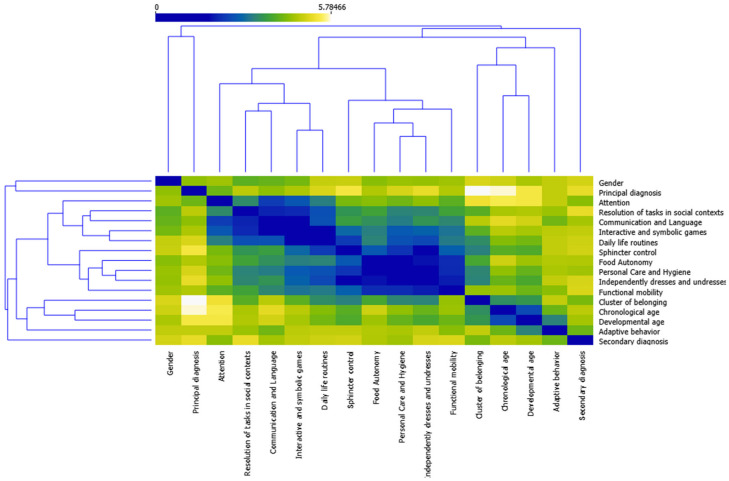
Results of application of hierarchical cluster with dendrogram modality.

The hierarchical clustering technique was also applied (this is based on the distance between each of the data, seeking to ensure that the data within a cluster are the most similar to each other) as it provides a graphical representation in dendrogram mode that allows the similarity between two objects to be visualized from an analysis of the height of the closest common node (see Figure 9).

**Figure 10:**
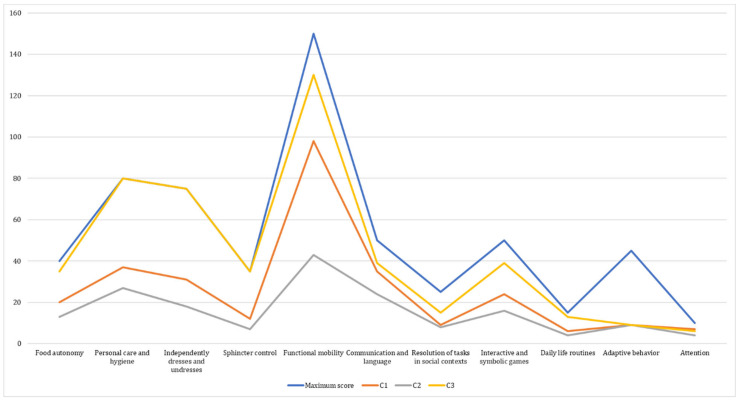
Comparison of scores in the 11 functional areas by cluster, and maximum scores for each area.

We used the mosaic display technique to better visualize the distribution as a function of the primary diagnosis, chronological age, and cluster variables. It allows us to visualize the peculiarities of the distribution in each area of functional development, which is essential for producing personalized therapeutic intervention programs (see Supplementary Materials Figure S1). In addition, the behavior of the clusters in terms of development in each of the functional areas is shown in Figure 10.

**Figure 11:**
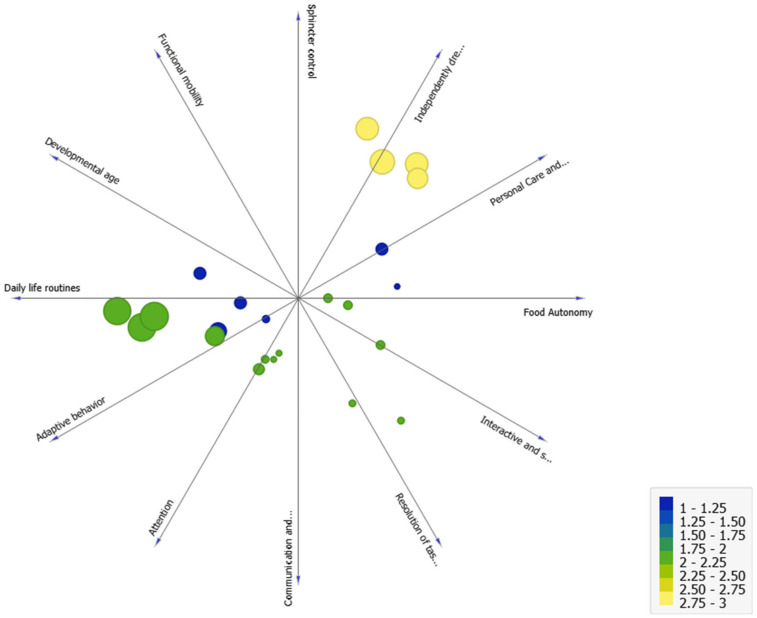
Analysis of cluster distribution in terms of the development age variable as a predictor of development in the different functional areas.

We also applied supervised machine learning techniques to examine prediction, specifically the multiple regression technique. We made a prediction based on the developmental age variable about functional development in the different areas. We found that developmental age predicted 75% of the development in the various areas (R^2^ = 0.75). This is illustrated in Figure 11 (the size and color of the circles identifies the cluster, the largest circles are cluster 3, the medium-sized circles are cluster 2, and the smallest circles are cluster 1). In short, Figure 11 represents the distribution of the clusters with respect to the development found in the different functional sub-areas measured by the eEarlyCare-T web application. The clustering predictor variable chosen was developmental age.

## 4. Discussion

Using technological tools such as computer applications makes it easier to treat and interpret results [16]. This study began with the creation of the eEarlyCare-T web application, specifically the module for recording and treating data from systematic observations of development of functional abilities in small children with various problems; see the works by Sáiz-Manzanares, Marticorena- Sánchez, Arnaiz-González, Díez-Pastor and García-Osorio [13] and Sáiz-Manzanares, Marticorena-Sánchez and Arnaiz-González [17]. This application allows data to be exported to statistical analysis software and allows the application of machine learning techniques [8]. More specifically, in this article, we presented the creation of the Therapeutic Program module in the eEarlyCare-T web application. This module uses the development profile from the Recording and treatment module to facilitate the creation of personalized therapeutic intervention programs using unsupervised learning techniques (clustering). This method of working is common in the treatment of oncological diseases [2,3,4,5], phobias, and depressive disorders [5,7], but the application to problems of development in young children is novel [8,9,10].

The design and implementation of these types of computer applications will make it easier to develop therapeutic interventions that are suitable for each user or group of users, which will likely lead to better intervention results and cost-effective use of personnel and material resources. The main results are about the creation of the two modules of the eEarlyCare-T web application, the Recording and Treatment of Data module, and the Therapeutic Intervention module. Both have a user-friendly interface which is easy for early care staff to use.

The limitations of this study relate to the fact that the eEarlyCare-T web application was tested with a small sample. Nonetheless, at the moment, the eEarlyCare-T research project is examining the participation of other groups and therapeutic care services for people with functional development problems. In future studies, there will be larger samples with a broader spread of characteristics. For these reasons, the results should be taken with caution, as only four therapists participated in this pilot study.

The eEarlyCare project began in 2018. It was selected as part of the sixth edition of the Proof of Concept competition (VI Convocatoria Prueba Concepto): Encouraging the assessment and commercialization of research results in which a first phase of application is done. In addition, the continuation of the project for the creation of the Therapeutic Program module was selected as part of the seventh edition of the Proof of Concept competition: Encouraging the assessment and exploitation of research results. Both were financed with European regional development funds (FEDER).

## 5. Conclusions

The development of web tools that facilitate the processing, interpretation and visualization of data collected in systematic observation processes in assessment settings for 0–6-year-olds opens the door to innovation for early care professionals (neonatologists, neurologists, pediatricians, physiotherapists, psychiatrists, psychologists, physiotherapists, speech therapists, etc.). These tools are expected to enable more accurate diagnoses. Likewise, the incorporation of learning analytics techniques within these tools will guide the creation of personalized intervention programs. All of this will improve the longitudinal monitoring of affected patients’ progress. In addition, we foresee that these tools will be useful in the distribution of personal and material resources, making them more cost effective. Ultimately, the use of these instruments is postulated as an aid to diagnostic adjustment and therapeutic intervention. However, in order to obtain conclusive data, comparative studies between traditional interventions and interventions with app-based tools are needed.

One of the strong points of this work is the use of web applications to record data in assessment and diagnosis processes. This functionality is relevant, as it will allow the application of different data analyses that, on the one hand, will provide the professional with information about the most affected area or areas in each user, and depending on this analysis, the most appropriate therapeutic intervention program for each user can be offered. The web application that has been presented contains this functionality. However, as weak points, this web application does not include automatic learning techniques correctly inserted in the application, so the data must be extracted and imported into programs that do include them. This can be a difficult challenge for some professionals unfamiliar with this type of analysis. Future research will attempt to address this challenge. This conclusion is directly related to that indicated by Kaelin et al. [21] in their recent systematic review study.

## 6. Patents

The web application “eEarly Care Therapeutic Intervention Program” is registered in the General Intellectual Property Registry (Registro General de la Propiedad Intelectual) in the Spanish Ministry of Culture and Sports, Number NºR 00/2021/1174. Exploitation rights are held by the University of Burgos. The application can be used under a license agreement with the University of Burgos.

## Figures and Tables

**Table 1:** Description of the sample characteristics.

Principal Diagnosis		Girls			Boys		
	N	Mage	SDage	n	Mage	SDage	n
1. Mild Intellectual Disability	2	-	-	0	54	24.46	2
2. Intellectual Disability Moderate	4	72	-	1	60	20.78	3
3. Severe Intellectual Disability	3	-	-	0	72	0	3
4. Global Developmental Delay	13	41.5		4	38.66	20.05	9
5. Dysphasia	1	-	-	0	40	-	1

Note. Mage: Mean age in month; SDage = Standard Deviation age.

**Table 2:** Closed response items from the usability questionnaire.

Closed Response Items	Type of Response	Score (1 to 5)					% Score				
		1	2	3	4	5	1	2	3	4	5
6. The eEarlyCare-T web application facilitates the recording of functional skills assessment results in young children.	Does not facilitate/ facilitates				1	3				33	67
7. The eEarlyCare-T web application facilitates the interpretation of the results of the assessment of functional skills in young children.	Does not facilitate/ facilitates				1	3				33	67
8. The design of the eEarlyCare-T web application is.	Unpleasant/ pleasant				1	3				33	67
9. Navigating the eEarlyCare-T web application is.	Unpleasant/ pleasant				1	3				33	67
10. The web application eEarlyCare-T is.	Hard to understand /intuitive				1	3				33	67
11. The customised intervention program in the eEarlyCare-T web application seems to be.	Very bad/ Very good					4					100
12. I found the eEarlyCare-T web application easy to use.	Never/ Always				1	3				33	67
13. I needed help to use the eEarlyCare-T web application.	Never/ Always	1					100				
14. The eEarlyCare-T web application facilitates the recording of functional skills assessment results in young children aged 0–6 years.	Never/ Always					4					100
15. The eEarlyCare-T web application facilitates the interpretation of the results of the assessment of functional skills in young children 0–6 years.	Never/ Always					4					100

Note. The items 1–5 refer to non-identifying personal data: 1 = Informed consent; 2 = Age; 3 = Gender; 4 = Level of education; % = Employment status.

**Table 3:** Open response items from the usability questionnaire.

Items (Open Response Items)	Answer Categorization
16. Would you recommend health professionals use the eEarlyCare web application? Why?	Yes, it makes the therapist’s job much easier
17. Describe the most important aspects of the eEarlyCare-T web application.	Allows the follow-up of children and the comparison of intra and inter-patient evolution.
18. What elements would you add to the eEarlyCare-T web application?	Help the therapist through an intelligent voice assistant and the export of the guidance given in the intervention program.
19. What elements would you remove from the eEarlyCare-T web application?	None

**Table 4:** Centers of final clusters in the 11 functional areas measured by SFA included in the eEarlyCare-T web application.

Functional Area Scores	Maximum Score	Centers of Final Clusters		
		1 n = 6	2 n = 13	3 n = 4
Food autonomy	40	20	13	35
Personal care and hygiene	80	37	27	80
Independently dresses and undresses	75	31	18	75
Sphincter control	35	12	7	35
Functional mobility	150	98	43	130
Communication and language	50	35	24	39
Resolution of tasks in social contexts	25	9	8	15
Interactive and symbolic games	50	24	16	39
Daily life routines	15	6	4	13
Adaptive behavior	45	9	9	9
Attention	10	7	4	6

Note. A high score indicates behavior problems; a low score indicates the absence of those problems.

**Table 5:** Cross table between participant’s cluster and primary diagnosis.

Primary Diagnosis	Cluster Case Number			Total
	1	2	3	
1. Mild Intellectual Disability	0	2	0	2
2. Intellectual Disability Moderate	0	1	3	4
3. Severe Intellectual Disability	1	2	0	3
4. Global Development Delay	4	8	1	13
5. Dysphasia	1	0	0	1

## Data Availability

The database of this pilot study may be requested upon written request from the university or institution that endorses that the data will be used for scientific purposes and after signing a data protection agreement with the data protection officer of the University of Burgos.

## Supplementary Materials

The following supporting information can be downloaded at: https://www.mdpi.com/article/10.3390/ijerph19116558/s1, Video S1: How it works: the eEarlyCare-T web application. Table S1: Questionnaire of the assessment usability of the eEarlyCare-T web application; Figure S1: Mosaic Display in the 11 functional areas of eEarlyCare-T web application.
