# Supplementary material for: Improvements for Therapeutic Intervention from the Use of Web Applications and Machine Learning Techniques in Different Affectations in Children Aged 0–6 Years
Source: Int J Environ Res Public Health. 2022 May 27;19(11):6558. doi: 10.3390/ijerph19116558 (PMC9180398; doi:10.3390/ijerph19116558)

International Journal of
Environmental Research and Public Health

MDPI

Supplementary Materials

Video S1. How it works the eEarlyCare web

https://bit.ly/3IOHx3A

Table S1. Questionnaire of the assessment usability of the eEarlyCare web application.

The data collected in this survey are not nominative and are only used for research purposes within the project of "eEarly Care Therapeutic Intervention Program". This project is co-financed by the Junta de Castilla y León and the European Union through FEDER funds to support the development of three proof concepts: Promotion of the valorisation and exploitation of research results. In accordance with Regulation (EU) 2016/679, General Data Protection Regulation, and O.L. 3/2018, of 5 December, on the Protection of Personal Data and guarantee of digital rights, we inform you that your personal data are processed by THE UNIVERSITY OF BURGOS for the purpose of managing and maintaining the professional relations that bind us with you. Your data may be transferred to the public entities and administrations necessary for the performance of such management and for the satisfaction of the legitimate interests pursued by both parties. This data processing is necessary to maintain this professional relationship. The data will be deleted at the end of the statute of limitation periods established by law, and will only be kept for the purpose of dealing with possible claims. You may exercise your rights of access, rectification, cancellation, opposition, portability and limitation of the processing of your data by contacting the General Secretariat of the University of Burgos CIF: Q-0968272-E Address: Hospital del Rey s/n. CP 09001 Burgos. Spain, enclosing a copy of your ID card. As a public administration, we apply the technical and organisational measures dictated by the National Security Scheme. This includes a series of recommendations to try to guarantee the security of information systems and thus avoid theft, alteration or unauthorised access to data. In the case of subcontracting services, we will demand and ensure that the data processor applies measures analogous to those of the National Security Scheme. In this case, you must provide supporting documentation:

▪ Accreditation of the identity of the data subject by means of any valid document, such as ID card or passport.
▪ Name and surname(s) of the interested party or, where applicable, of the person representing him/her, as well as the document accrediting such representation.
▪ The request which is the subject of the application.
▪ Address for service, date and signature of the applicant.
▪ Documents supporting the request you are making, if applicable.

In the case of rectification or cancellation, indication of the data to be rectified or cancelled and the reason that justifies it. In the event that you wish to file a complaint or obtain additional information on the regulation of the processing of personal data in Spain, the competent authority is the Spanish Data Protection Agency (C/ Jorge Juan, 6 28001-Madrid (Spain).

Personal Information

Mark with a cross the answer that relates to your personal information

1. I have been informed about the objectives of the project "eEarly Care" and data processing. I can at any time express my disagreement to participate in this project. Therefore, I agree to participate in this opinion survey Yes or No

2. Age

a) 20-24; b) 25-29; c) 30-34; d) 35-39; d) 40-44; e) 45-49; f) More than 50

3. Gender

a) Men; b) Woman; c) No answer

4. Educational level

a) Bachelor degree; b) Master degree; c) Doctorate

5. Employment situation

a) Unemployed; b) On the job

**Closed-answer questions** (Next is a series of questions regarding your perception of the usability of the eEarlyCare web application, the answers are measured on a Likert-type scale from 1 (strongly disagree) to 5 (strongly agree). This survey is an adaptation of the User Experience Questionnaire https://www.ueq-online.org/ by Laugwitz, Schrepp, & Held (2008).	**Rating scale**

**6.** The use of the eEarlyCare web application facilitates the recording of functional skills assessment results in young children.	1	2	3	4	5
**7.** The use of the eEarlyCare web application facilitates the interpretation of the results of the assessment of functional skills in young children.	1	2	3	4	5
**8.** The design of the eEarlyCare web application appears to be (1 Unpleasant-5 Pleasant).	1	2	3	4	5
**9.** Navigating the eEarlyCare web application seems to be (1 Unpleasant-5 Pleasant).	1	2	3	4	5
**10.** The web application "eEarly Care Therapeutic Intervention Program" seems to be (1 Hard understand-5 Intuitive).	1	2	3	4	5
**11.** The customised intervention program in the eEarlyCare web application seems to be (1 Very bad-5 Very Good).	1	2	3	4	5
**12.** I have found the eEarlyCare web application easy to use (1 Never-5 Always).	1	2	3	4	5
**13.** I needed help using the eEarlyCare web application (1 Never-5 Always).	1	2	3	4	5
**14.** The use of the eEarlyCare web application facilitates the recording of functional skills assessment results in young children 0-6 years (1 Never-5 Always).	1	2	3	4	5
**15.** The eEarlyCare web application facilitates the interpretation of the results of the assessment of functional skills in young children 0-6 years.	1	2	3	4	5
**Open-ended questions**					
**16.** Would you recommend the use of the eEarlyCare web application in the work of the health professional? Why?					
**17.** Describe the most relevant aspects of the eEarlyCare web application.					
**18.** What elements would you include in the eEarlyCare web application?					
**19.** What elements would you remove from the eEarlyCare web application?					

Figure S1. Mosaic Display in the 11 functional areas of eErlyCare web application.

Food Autonomy Functional Area	Personal care and hygiene Functional Area

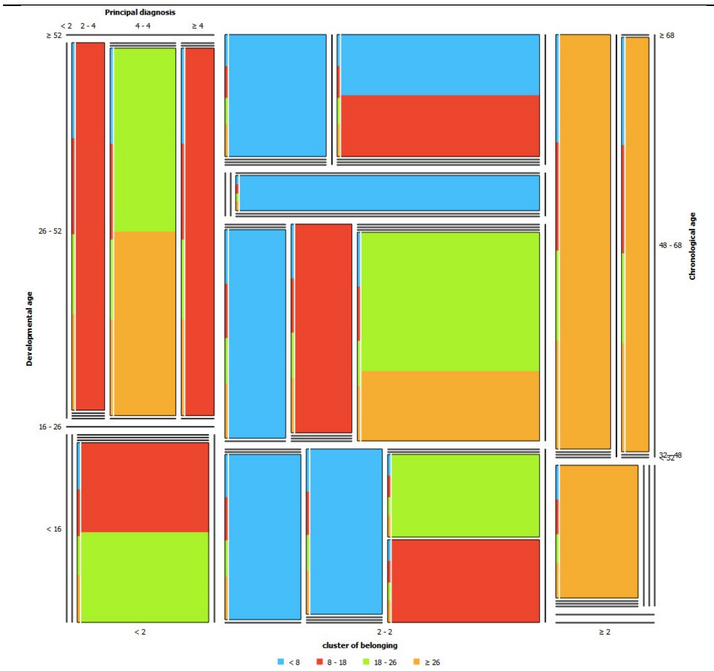

Independently dresses and undresses Functional Area

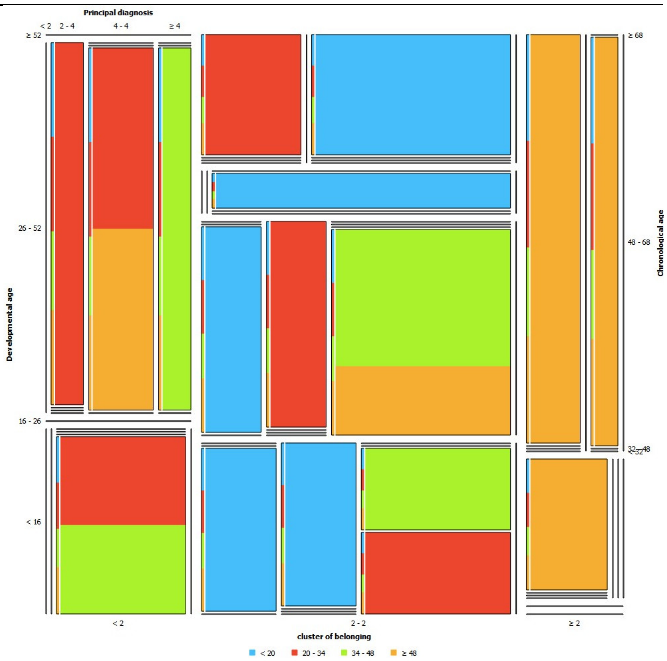

Sphincter control Functional Area

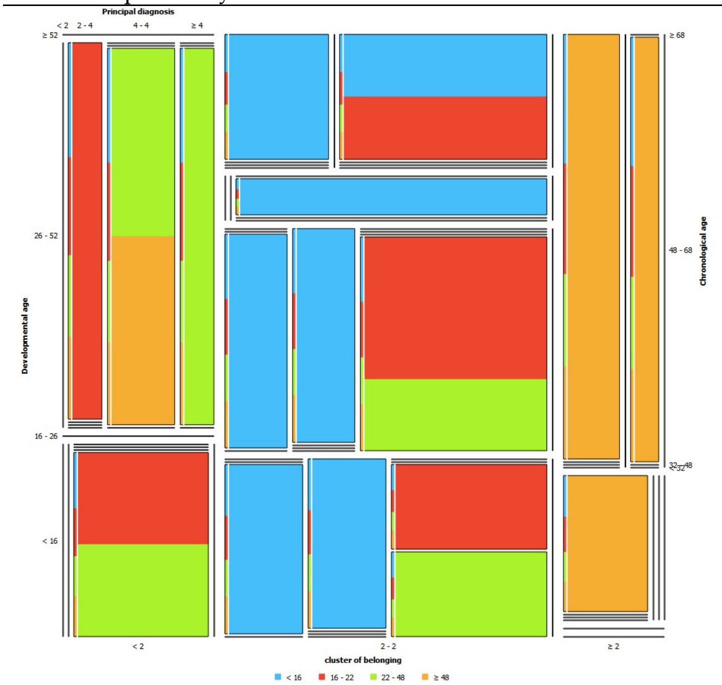

Functional mobility Functional Area

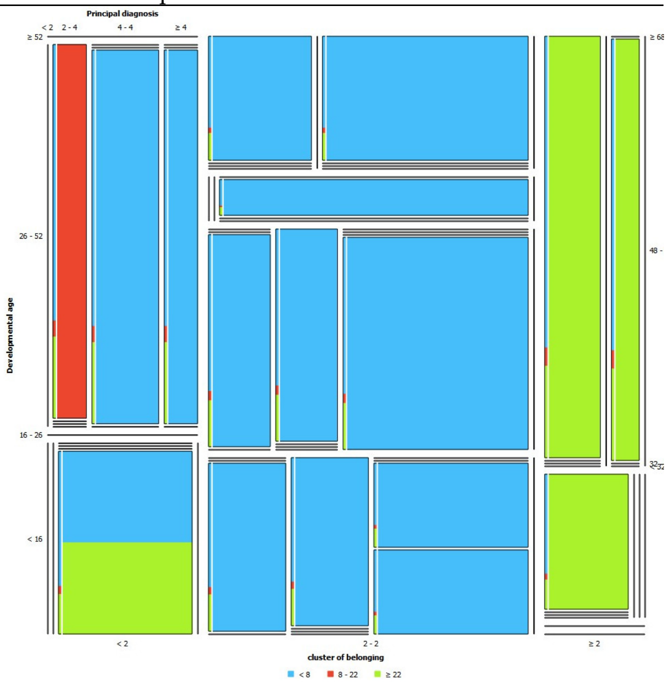

Communication and Language Functional Area

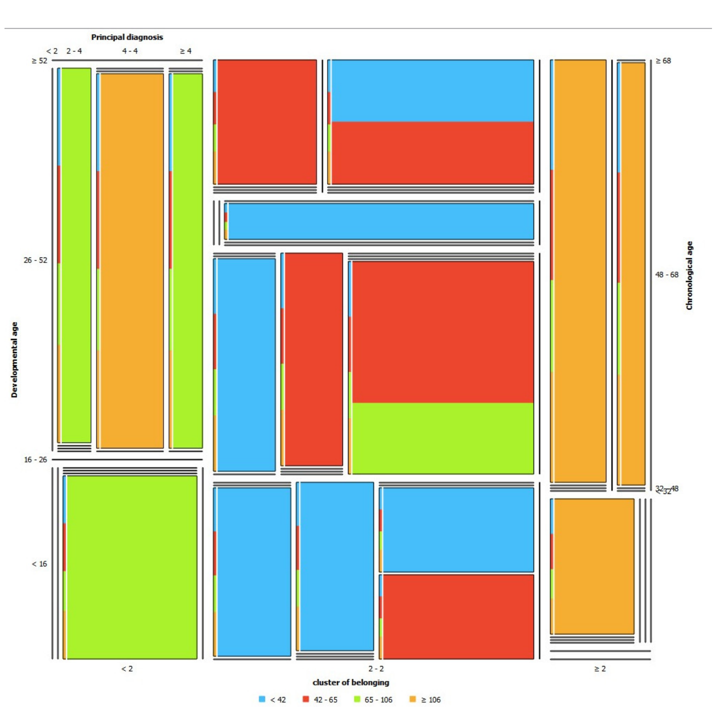

Resolution of tasks in social contexts Functional Area

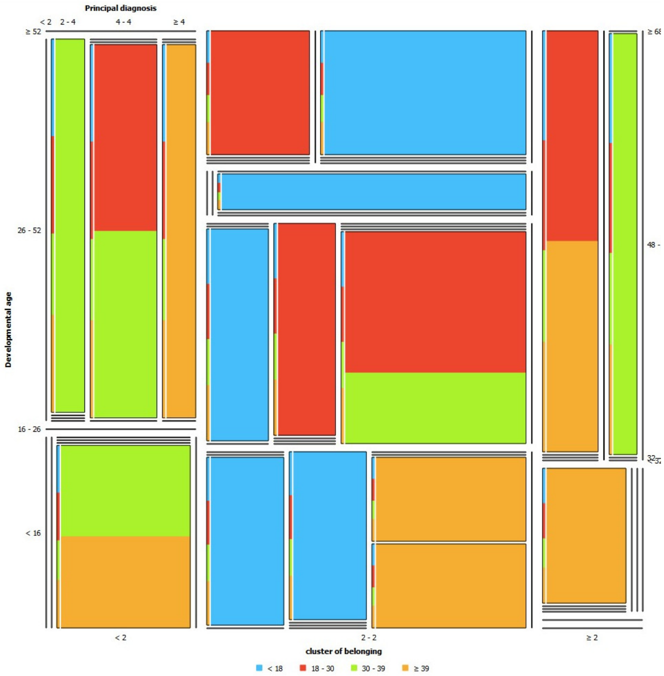

Interactive and symbolic games Functional Area

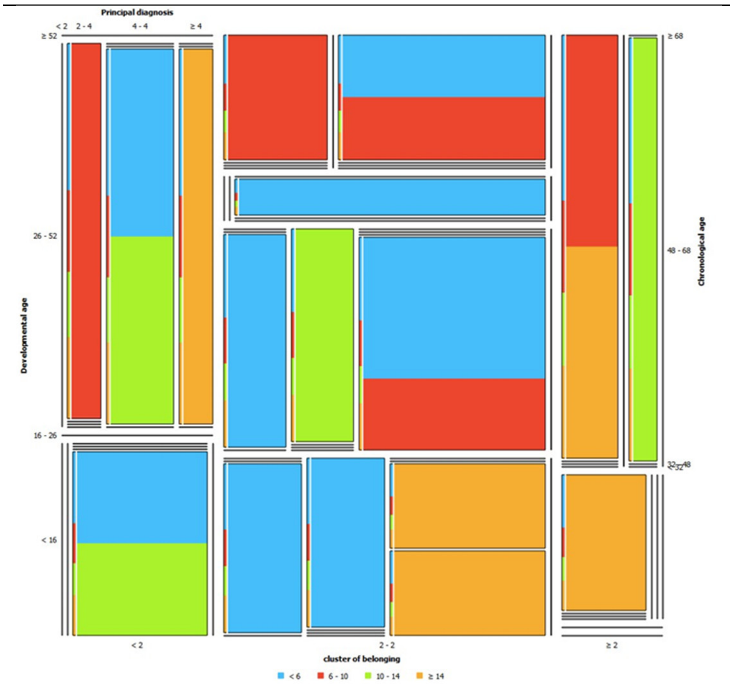

Daily life routines Functional Area

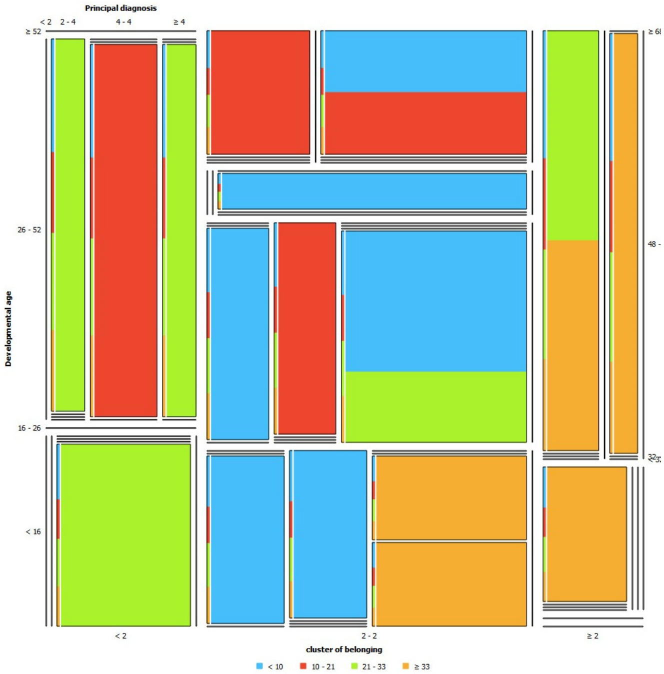

Adaptive behavior Functional Area

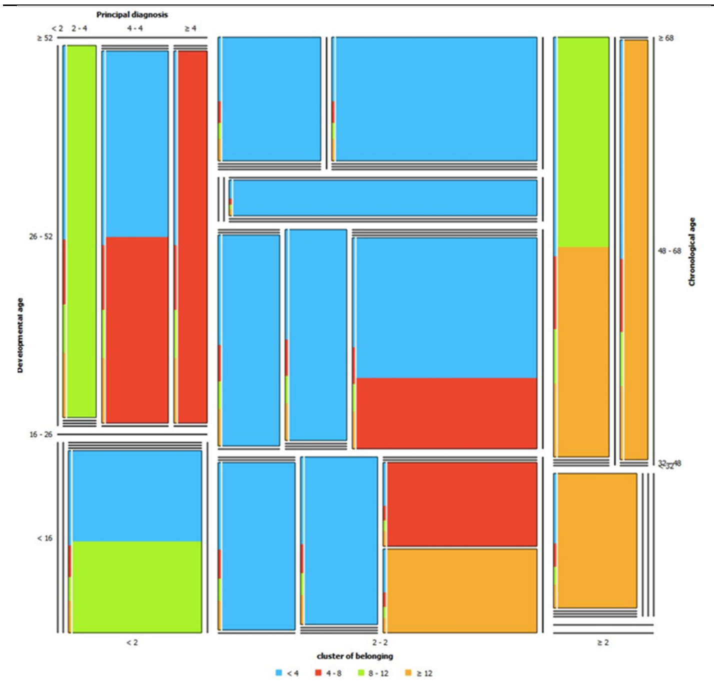

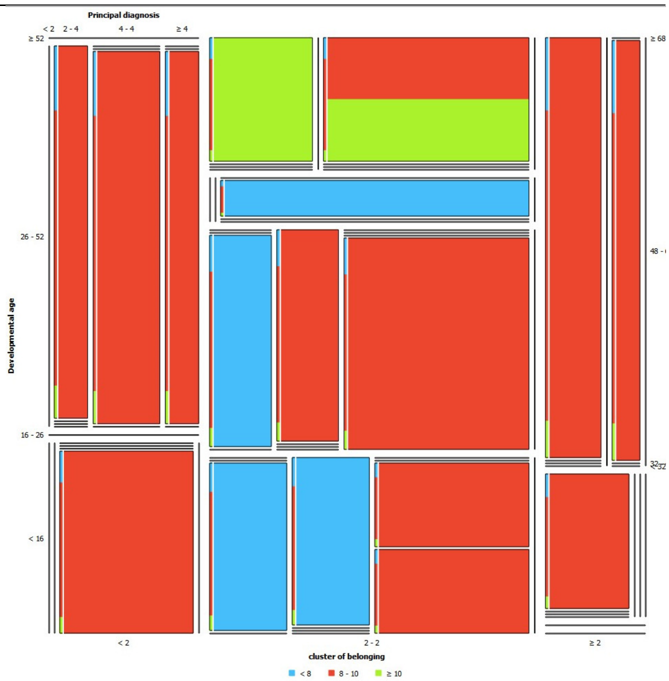

Attention Functional Area

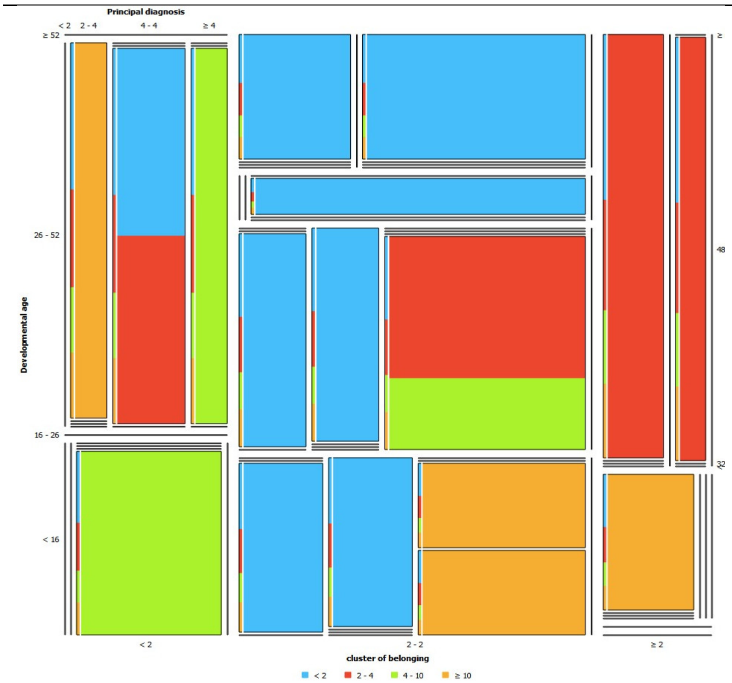

Supplement: Supplementary file 1 [file ijerph-19-06558-s001.zip › ijerph-1668701-supplementary.pdf]
